# A peak in the critical current for quantum critical superconductors

**DOI:** 10.1038/s41467-018-02899-5

**Published:** 2018-01-30

**Authors:** Soon-Gil Jung, Soonbeom Seo, Sangyun Lee, Eric D. Bauer, Han-Oh Lee, Tuson Park

**Affiliations:** 1 Center for Quantum Materials and Superconductivity (CQMS), Department of Physics, Sungkyunkwan University, Suwon, 16419 South Korea; 2 Los Alamos National Laboratory, Los Alamos, NM 87545 USA; 3 Center for Correlated Matter and Department of Physics, Zhejiang University, 310058 Hangzhou, China

**Keywords:** Electronic properties and materials, Superconducting properties and materials

## Abstract

Generally, studies of the critical current *I*_c_ are necessary if superconductors are to be of practical use, because *I*_c_ sets the current limit below which there is a zero-resistance state. Here, we report a peak in the pressure dependence of the zero-field *I*_c_, *I*_c_(0), at a hidden quantum critical point (QCP), where a continuous antiferromagnetic transition temperature is suppressed by pressure toward 0 K in CeRhIn_5_ and 4.4% Sn-doped CeRhIn_5_. The *I*_c_(0)s of these Ce-based compounds under pressure exhibit a universal temperature dependence, underlining that the peak in zero-field *I*_c_(*P*) is determined predominantly by critical fluctuations associated with the hidden QCP. The dc conductivity *σ*_dc_ is a minimum at the QCP, showing anti-correlation with *I*_c_(0). These discoveries demonstrate that a quantum critical point hidden inside the superconducting phase in strongly correlated materials can be exposed by the zero-field *I*_c_, therefore providing a direct link between a QCP and unconventional superconductivity.

## Abstract (web summary)

Knowledge of critical current may provide important information to understand unconventional superconductivity and quantum critical behavior. Here, Jung et al. observe a peak in the pressure dependence of the zero-field critical current at a hidden quantum critical point in pure and Sn-doped heavy fermion superconductor CeRhIn_5_.

## Introduction

Unconventional superconductivity (SC) often is observed in close proximity to a magnetically ordered phase, where the SC transition temperature *T*_c_ forms a dome against a non-thermal control parameter, such as the external pressure, chemical substitution, or magnetic field^1–6^. At an optimal value of the tuning parameter, where *T*_c_ is the highest, normal state properties do not follow predictions for Landau–Fermi liquids: the electrical resistivity (*ρ*) does not exhibit a *T*^2^ dependence, and the electronic specific heat coefficient (*γ*  *=* *C*/*T*) does not saturate, but rather diverges with decreasing temperature^1, 2, 7^. These non-Fermi liquid (NFL) behaviors arise from incoherent critical fluctuations associated with a quantum critical point (QCP) hidden inside the SC dome of heavy fermion compounds and some Fe-based superconductors, such as BaFe_2_(As_1−*x*_P_*x*_)_2_^1, 2, 4, 6, 8^. Because the zero-temperature quantum phase transition is typically not accessible without destroying SC, the role of critical magnetic fluctuations on properties of unconventional superconductors has yet to be explored in depth.

The critical current (*I*_c_), which limits the current capacity of a zero-resistance state, is characteristically taken to depend on the strength of vortex pinning, which, in turn, is determined by the geometry and distribution of microstructural defects^9–11^. Because application of pressure should not lead to the creation of different or additional defects or to a substantial change in sample dimensions, *I*_c_ in relation to *T*_c_ should be at most weakly pressure-dependent. A substantial variation in *I*_c_(*P*) or *I*_c_/*T*_c_(*P*), then, logically, should be attributed to intrinsic changes in the superconducting state itself. For example, the zero-field critical current density *J*_c_ (equal to *I*_c_/*A*, where *A* is the sample cross sectional area perpendicular to current) of the hole-doped high-*T*_c_ cuprate superconductor Y_0.8_Ca_0.2_Ba_2_Cu_3_O_*y*_ has a sharp peak that is centered on a critical hole-doping where the pseudogap boundary line projects to zero temperature, and that is attributed in model calculations to changes in the superfluid density^12, 13^. These results indicate that *I*_c_ measurements may provide an opportunity to explore the relationship between unconventional SC and any QCP that is hidden beneath the SC dome.

Here we report a peak in the zero-field critical current, *I*_c_(0), at a critical pressure *P*_c_ in pure CeRhIn_5_ (Rh115) and 4.4% Sn-doped CeRhIn_5_ (SnRh115), where their respective antiferromagnetic boundary *T*_N_(*P*) extrapolates to *T* = 0 K inside a dome of pressure-induced SC. The temperature dependence of *I*_c_(0)s for pure Rh115 and SnRh115 under pressure is similar to that of superconducting CeCoIn_5_, which is close to quantum criticality at ambient pressure. Normalized values of *I*_c_(*T*,* P*) follow a common universal curve for each material, suggesting an intrinsic, fundamental connection to quantum criticality. Supporting this conclusion, the magnetic field dependence of the flux-pinning force (*F*_p_ = *I*_c_ × *μ*_0_*H*), normalized to its maximum value, also forms a pressure-invariant universal curve for each compound. As will be discussed, these discoveries demonstrate that the pressure evolution of zero-field *I*_c_ is determined mainly by quantum critical fluctuations, and that the peak in *I*_c_ is a direct link to the hidden QCP.

## Results

### Temperature–pressure phase diagrams

**Fig. 1:**
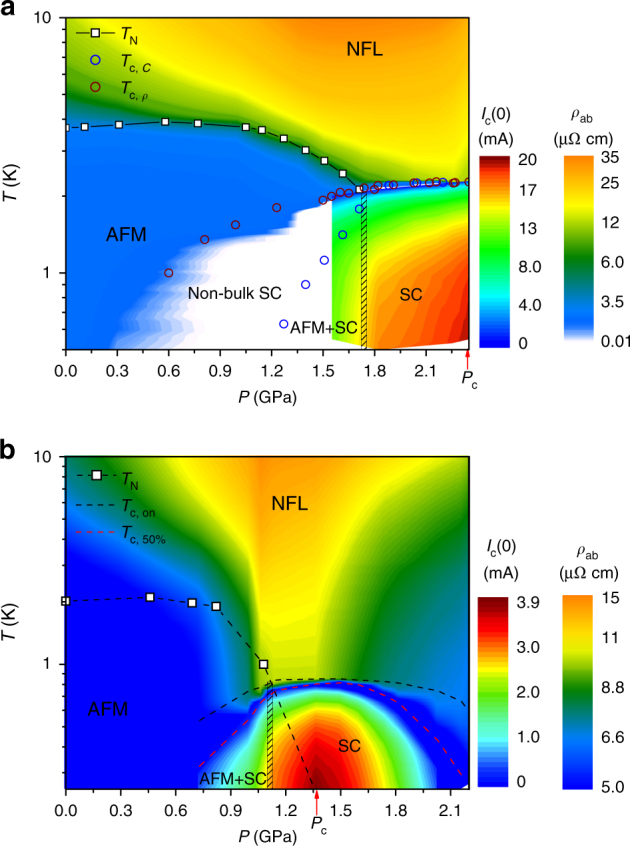
Temperature–pressure phase diagrams of CeRhIn_5_ and CeRhSn_0.22_In_4.78_ single crystals. In the superconducting state below *T*_c_(*P*), false colors denote the magnitude of the zero-field critical current *I*_c_(*P, T*). At temperatures above *T*_c_(*P*), false colors reflect the magnitude of the in-plane resistivity *ρ*_ab_(*P*, *T*). **a** CeRhIn_5_ (Rh115) and **b** CeRhSn_0.22_In_4.78_ (SnRh115). For both materials, *ρ*_ab_(*P*, *T*) is enhanced around the quantum critical point *P*_c_ due to pronounced incoherent inelastic scattering. Similarly, the zero-field *I*_c_(*P*, *T*) is largest at *P*_c_, where the QCP is expected, as indicated by the arrow. In both **a** and **b** the vertical hashed rectangle is at *P*_c_*, the pressure that separates a phase of coexisting superconductivity and magnetism from a purely SC phase for *P* > *P*_c_*. Open squares in both **a** and **b** represent the antiferromagnetic transition temperature (*T*_N_). SC transition temperature (*T*_c_) of Rh115 is evaluated from specific heat (*T*_c,__*C*_) and resistivity (*T*_c,__*ρ*_) measurements, and *T*_c_ of SnRh115 is determined as *T*_c_ onset (*T*_c,on_) and 50% (*T*_c,50%_) of the normal state resistivity value at *T*_c,on_. AFM, SC, and NFL stand for antiferromagnetic, superconducting, and non-Fermi liquid regions, respectively

Figure 1a and b presents a contour plot of the zero-field *I*_c_(*P*,* T*) in the SC phase and the in-plane resistivity *ρ*_ab_(*P, T*) in the normal state for pure CeRhIn_5_ (Rh115) and 4.4% Sn-doped CeRhSn_0.22_In_4.78_ (SnRh115) single crystals. The dependence on pressure of the in-plane resistivity and current–voltage curves upon which Fig. 1 is based is displayed in Supplementary Figs. 1 and 2, respectively. The quantum critical region veiled by the superconducting phase is fully exposed by the pressure dependence of zero-field *I*_c_(*T*). A sharp peak in the value of *I*_c_(*T*) is clearly observed for pressures around the QCP at *P*_c_, where a large enhancement in the resistivity is accompanied by strong quantum fluctuations^3,4,14^. In addition, *I*_c_(*P*) abruptly increases at pressures around *P*_c_*, the critical pressure where coexisting phases of magnetism and SC evolve into a single SC state. In undoped Rh115, large differences between *T*_c_s measured by heat capacity (*C*) and resistivity (*ρ*) at pressures below *P*_c_* are ascribed to textured SC originating from an incommensurate long-range magnetic order^15–18^.

### Temperature dependences of the zero-field critical current

**Fig. 2:**
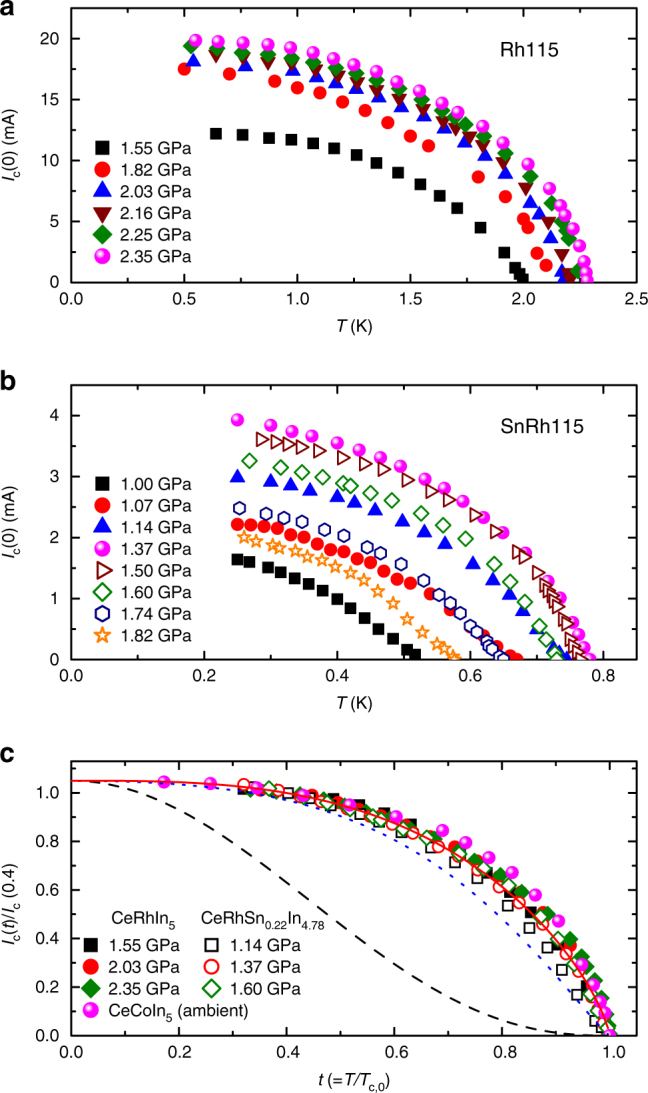
Temperature dependences of the zero-field critical current for Ce-based heavy fermion materials under pressure. **a** Temperature dependence of the zero-field critical current, *I*_c_(0), for CeRhIn_5_ at various pressures. **b** Zero-field *I*_c_ for CeRhSn_0.22_In_4.78_ at various pressures. **c** Reduced temperature (*t *= *T*/*T*_c,0_) dependence of *I*_c_, *I*_c_(*t*), normalized by its value at *t* = 0.4 for Rh115 and SnRh115 at representative pressures and for CeCoIn_5_ at ambient pressure. The normalized values of *I*_c_(*P*,* t*) for all crystals can be described by a single curve, *I*_c_(*t*) ∝ (1 − *t*^2^)^5/6^(1 + *t*^2^)^2/3^, indicating universal behavior of *I*_c_(*t*) with respect to pressure in the Ce*M*In_5_ (*M* = Co, Rh) materials. Dotted and dashed curves are for *δT*_c_-pinning and *δl*-pinning, respectively, as discussed in the text

The antiferromagnetic transition temperature (*T*_N_ ~ 3.8 K) in pure Rh115 is suppressed by Sn doping, which induces a shift of its extrapolated *T* = 0 K antiferromagnetic transition, and pressure-induced superconductivity emanates from the tuned QCP^4,5^ (see Supplementary Fig. 3). Figure 2a and b shows the temperature dependence of the zero-field critical current, *I*_c_(0), for Rh115 and SnRh115 at several pressures, respectively. Here, *I*_c_ is determined by using the voltage criterion of 0.1 μV (see Supplementary Fig. 2). Analysis of the flux-pinning force, *F*_p_ = *I*_c_ × *μ*_0_*H*, shows that the normalized flux-pinning force follows a power-law dependence on magnetic field, *f*_p_(*h*) ∝ *h*^*p*^(1−*h*)^*q*^, and is peaked around *h*_peak_ ≈ 0.6, which is characteristic of type-II superconductors with weak pinning (see Supplementary Fig. 4). Here, the normalized pinning force is *f*_p_ = *F*_p_/*F*_p,max_ and the reduced field is *h* = *H/H*_irr_, where *F*_p,max_ is the maximum flux-pinning force and *H*_irr_ is the irreversible field. The dependence on temperature of the critical current has been widely explained by *I*_c_(*t*)/*I*_c_(0) = (1−*t*^2^)^*α*^(1 + *t*^2^)^*β*^ for type-II superconductors, such as high-*T*_c_ cuprates, Fe-based superconductors, and MgB_2_^19–22^, where *t *=* T*/*T*_c_ is the reduced temperature. When *T*_c_ variations surrounding defects are important (*δT*_c_-pinning), *α* = 7/6 and *β* = 5/6, but *α* = 5/2 and *β* = −1/2 for *δl*-pinning that arises from spatial variations in the charge-carrier mean free path (*l*) near a lattice defect^10, 19, 23^. These functional forms are shown by the dotted and dashed lines for *δT*_c_-pinning to *δl*-pinning in Fig. 2c, respectively. A crossover of the mechanism from *δT*_c_-pinning to *δl*-pinning has been often reported by introducing additional defects via chemical substitution or heavy ion irradiation, indicating that *δT*_c_-pinning is preferred in clean crystals^19–21^.

The values of *I*_c_(*t*) for SnRh115 at pressures around *P*_c_ are fitted together with those for Rh115 and CeCoIn_5_ in Fig. 2c. The temperature dependence of *I*_c_(0) for CeCoIn_5_ at ambient pressure is measured to compare it with that of CeRhIn_5_, because Rh115 is believed to have a SC pairing mechanism similar to that in CeCoIn_5_. The values of *I*_c_(0) for all samples can be expressed well by one curve with the relation *I*_c_(*t*) ∝ (1−*t*^2^)^5/6^(1 + *t*^2^)^2/3^, which is distinct from that for *I*_c_(*t*) controlled by either *δT*_c_-pinning or *δl*-pinning. This universal curve underscores that the origin of the zero-field *I*_c_ is the same for each compound, and that it does not change under pressure for these Ce-based quantum critical materials. The fact that external pressure does not create new defects inside the crystals suggests that the pressure evolution of *I*_c_ should be related to the pressure dependence of the SC coupling strength.

## Discussion

**Fig. 3:**
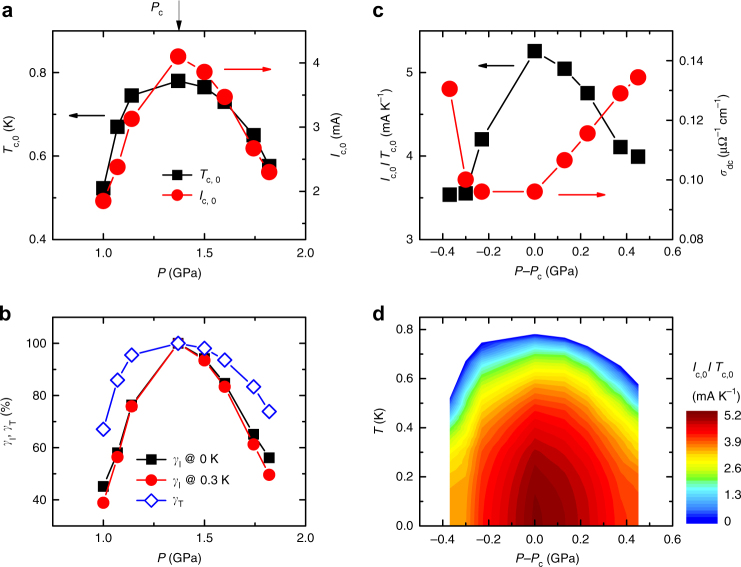
Pressure evolution of the zero-field critical current in Sn-doped CeRhIn_5_. **a** Pressure dependences of *T*_c,0_ and *I*_c,0_ for SnRh115, where *I*_c,0_ is the value of *I*_c_ obtained from an extrapolation of data in Fig.  2b zero Kelvin. **b** Fractional variations in *I*_c,0_ and *T*_c,0_ for SnRh115 under pressure. The fractions are defined as *γ*_*I*_ (at 0 K) ≡ *I*_c,0_(*P*)/*I*_c,0_(*P*_c_) × 100 and *γ*_*T*_ ≡ *T*_c,0_(*P*)/*T*_c,0_(*P*_c_) × 100, where *I*_c,0_(*P*_c_) is *I*_c_ extrapolated to zero temperature at *P*_c_ and *T*_c,0_(*P*_c_) is the superconducting transition temperature at *P*_c_. Values of *γ*_*I*_ are plotted as a function of pressure for measured or estimated *I*_c_(*T*, *P*) at 0 K (squares) and 0.3 K (circles). **c** The ratio between the critical current and SC transition temperature, *I*_c,0_/*T*_c,0_, plotted together with the dc conductivity at *T*_c_ onset, *σ*_dc_, as a function of the pressure difference *P*−*P*_c_, where *P*_c_ = 1.35 GPa is the QCP. **d** A contour plot of *I*_c,0_/*T*_c,0_ displayed in the temperature (*T*) and pressure (*P*−*P*_c_) plane. The ratio *I*_c,0_/*T*_c,0_ forms a dome centered around the quantum critical point *P*_c_ and its values decrease with distance from *P*_c_

Figure 3a presents the pressure dependences of *I*_c_(0) and *T*_c,0_ for SnRh115, which are similar to each other. However, their relative fractional variations in *I*_c_ and *T*_c_, *γ*_*I*_ ≡ *I*_c,0_(*P*)/*I*_c,0_(*P*_c_) × 100 and *γ*_*T*_ ≡ *T*_c,0_(*P*)/*T*_c,0_(*P*_c_) × 100, where *I*_c,0_(*P*_c_) is *I*_c_ extrapolated to zero temperature at *P*_c_ and *T*_c,0_(*P*_c_) is the SC transition temperature at *P*_c_, are much different, as shown in Fig. 3b: at 1.0 GPa, the critical current is 45% of the maximum value, and *T*_c,0_ is 67% of its maximal value. The stronger pressure dependence of *I*_c_ relative to that of *T*_c,0_ is clearly visible in ratio *I*_c,0_/*T*_c,0_ for SnRh115, as presented in Fig. 3c, d. An abrupt enhancement in *I*_c,0_/*T*_c,0_ is observed at *P*_c_*, and the peak in the pressure dependence of *I*_c,0_/*T*_c,0_ is achieved at *P*_c_. The dc conductivity (=*σ*_dc_) at *T*_c_ onset is shown as a function of pressure in the right ordinate of Fig. 3c, where a minimum value appears near *P*_c_ (see Supplementary Fig. 5). The anti-correlation between *I*_c,0_/*T*_c,0_ and *σ*_dc_ in these Ce-based quantum critical compounds may be related with the presence of the hidden QCP at *P*_c_, because the associated critical quantum fluctuations not only act as the SC pairing glue, but also strongly enhance incoherent electron scattering, thus leading to a minimum in *σ*_dc_ at *P*_c_^24, 25^. Homes’ scaling relation^26–28^ states that the superfluid density *n*_s_ is proportional to *σ*_dc_*T*_c_ in many correlated superconductors and, consequently, that the ratio *n*_s_/*T*_c_ should be proportional to *σ*_dc_. The fact that *σ*_dc_ is the minimum at *P*_c_, where *I*_c,0_/*T*_c,0_ is the maximum in these Ce-based compounds, suggests a violation of Homes’ scaling if the strength of the condensate *n*_s_ is proportional to the critical current *I*_c,0_. Pressure-dependent optical conductivity and/or penetration depth experiments that directly measure *n*_s_ will be important to provide a stringent test for the validity of Homes’ law in quantum critical superconductors.

Our study demonstrates that the critical current, a fundamental superconducting parameter, is a powerful tool for investigating the presence of a hidden QCP inside the superconducting dome without destroying the superconducting phase. The dependence on temperature of the zero-field *I*_c_ for both pure Rh115 and Sn-doped Rh115 exhibits the same functional form under pressure, underscoring that the peak at *P*_c_ in the pressure dependence of *I*_c_ arises from an enhanced fluctuations around the hidden QCP. Even though these results are specific to the Ce115 heavy-fermion materials, the prediction of similar results for the hole-doping dependence of the critical current density *J*_c_(*x*) in high-*T*_c_ cuprates^29^ suggests a universal behavior of *J*_c_ among unconventional superconductors. These discoveries should stimulate more theoretical and experimental effort to understand the intimate link between quantum criticality and the origin of unconventional superconductivity in various families of correlated electronic systems.

## Methods

### Measurement outline

CeRhIn_5_, Sn-doped CeRhIn_5_, and CeCoIn_5_ single crystals were synthesized by the indium (In) self-flux method^30–32^. Pressure was generated in a hybrid clamp-type pressure cell with Daphne 7373 as the pressure-transmitting medium, and the pressure was determined by monitoring the shift in the value of *T*_c_ for lead (Pb). Measurements of current–voltage (*I*–*V*) characteristics under pressure were performed in a Heliox VL system (Oxford Instruments) with a vector magnet *(y* = 5 T and *z* = 9 T, American Magnetics Inc.) and in a Physical Property Measurement System (PPMS 9 T, Quantum Design), where the current was provided by a Keithley 6221 unit and the voltage was measured with a Keithley 2182A nanovoltmeter.

### Measurement details

Measurements of *I–V* characteristics were performed in a pulsed mode to minimize Joule heating developed at Ohmic contacts to the samples and copper (Cu) wires between the pressure cell and the connector. The duration of the pulsed current was 10–11 ms, and the repetition rate was one pulse every 2 s, which was sufficient to eliminate Joule heat in the samples^33, 34^. A standard four-probe method was used to determine *I*−*V*, and good Ohmic contact to samples was achieved by using silver epoxy. The critical current was based on a 10^−7^ V criterion^35^, which was averaged over three measurements. The dimensions of the measured crystals were 920×330×20, 650×200×22, and 1100×200×47 μm^3^ for CeRhIn_5_ (Rh115), CeRhSn_0.22_In_4.78_ (SnRh115), and CeCoIn_5_, respectively. The magnetic-field dependence of the critical current was measured at several pressures and the flux-pinning force (*F*_p_) was estimated from the relation *F*_p_ = *I*_c_ × *μ*_0_*H* ^36–38^

### Data availability

The data sets generated and/or analyzed in this study are available from the corresponding author on reasonable request.

## Electronic supplementary material

Supplementary Information
