# Author Correction: A peak in the critical current for quantum critical superconductors

**DOI:** 10.1038/s41467-019-11621-y

**Published:** 2019-08-06

**Authors:** Soon-Gil Jung, Soonbeom Seo, Sangyun Lee, Eric D. Bauer, Han-Oh Lee, Tuson Park

**Affiliations:** 1 Center for Quantum Materials and Superconductivity (CQMS), Department of Physics, Sungkyunkwan University, Suwon, 16419 South Korea; 2 Los Alamos National Laboratory, Los Alamos, NM 87545 USA; 3 Center for Correlated Matter and Department of Physics, Zhejiang University, 310058 Hangzhou, China

**Keywords:** Electronic properties and materials, Superconducting properties and materials

Correction to: *Nature Communications* 10.1038/s41467-018-02899-5, published online 30 January 2018.

The original version of this Article contained an error in the author affiliations. The affiliation of Soonbeom Seo with ‘Los Alamos National Laboratory, Los Alamos, NM 87545, USA’ was inadvertently omitted. Additionally the original version omitted the following from the Acknowledgements: ‘S.S. acknowledges a Director's Postdoctoral Fellowship through the Los Alamos Laboratory Directed Research and Development program.’ This has now been corrected in both the PDF and HTML versions of the Article.

