# Supplementary material for: A peak in the critical current for quantum critical superconductors
Source: Nat Commun. 2018 Jan 30;9:434. doi: 10.1038/s41467-018-02899-5 (PMC5789853; doi:10.1038/s41467-018-02899-5)
Supplement: Supplementary file 1 — Supplementary Information [file 41467_2018_2899_MOESM1_ESM.pdf]

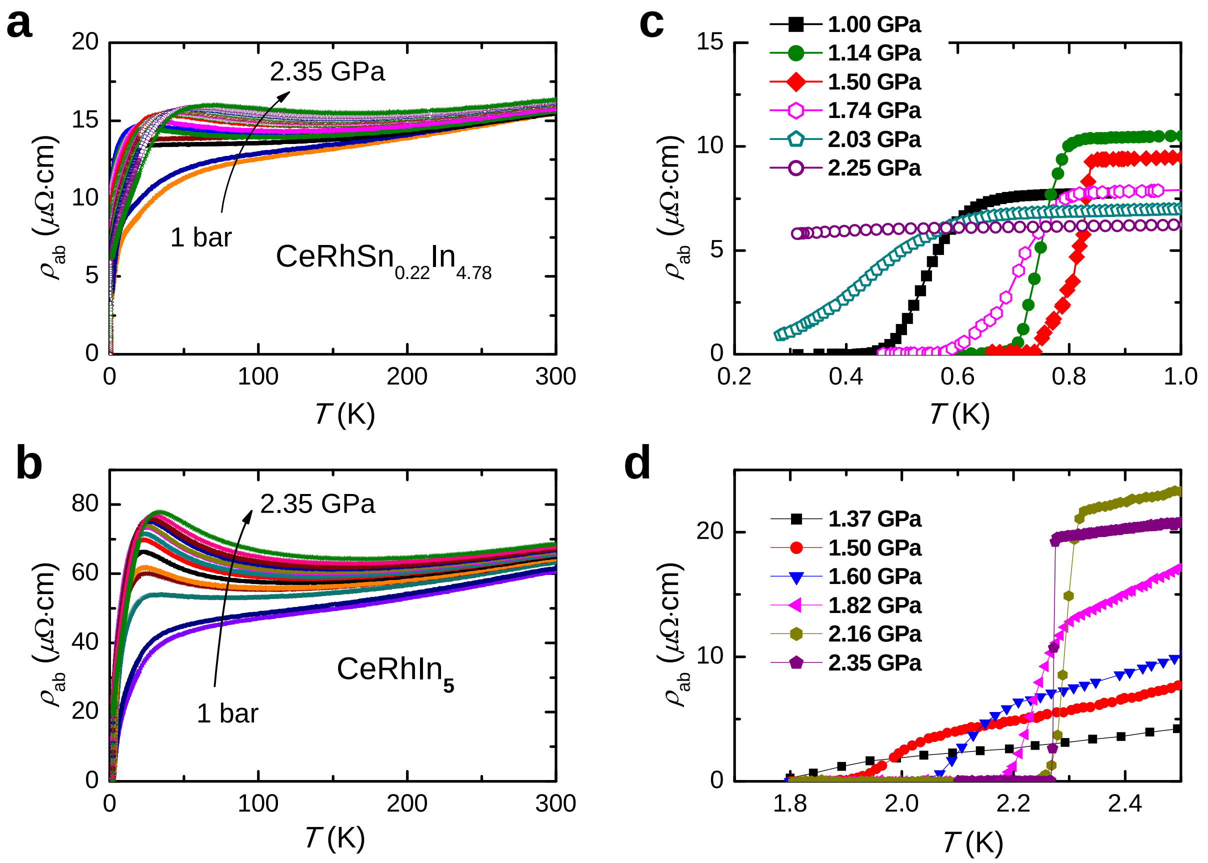

Supplementary Figure 1: Temperature dependences of the in-plane resistivity for $CeRhSn_{0.22}In_{4.78}$ and $CeRhIn_5$ under pressure. a, In-plane resistivity (ρ_{ab}) of the Sn-doped Rh115 (SnRh115) is plotted as a function of temperature for pressures, $\rho_{ab}(P,T)$, of 0, 0.14, 0.97, 1.0, 1.07, 1.14, 1.37, 1.5, 1.6, 1.74, 1.82, 2.03, 2.16, 2.25, and 2.35 GPa along the arrow direction. **b,** $\rho_{ab}(P,T)$ of undoped Rh115 is displayed against temperature for pressures of 0, 0.14, 0.97, 1.07, 1.14, 1.37, 1.5, 1.6, 1.74, 1.82, 2.03, 2.16, 2.25, and 2.35 GPa along the arrow direction. A magnified view of $\rho_{ab}(P,T)$ near the SC transition temperatures is shown at selected pressures for SnRh115 and Rh115 in **c** and **d**, respectively.

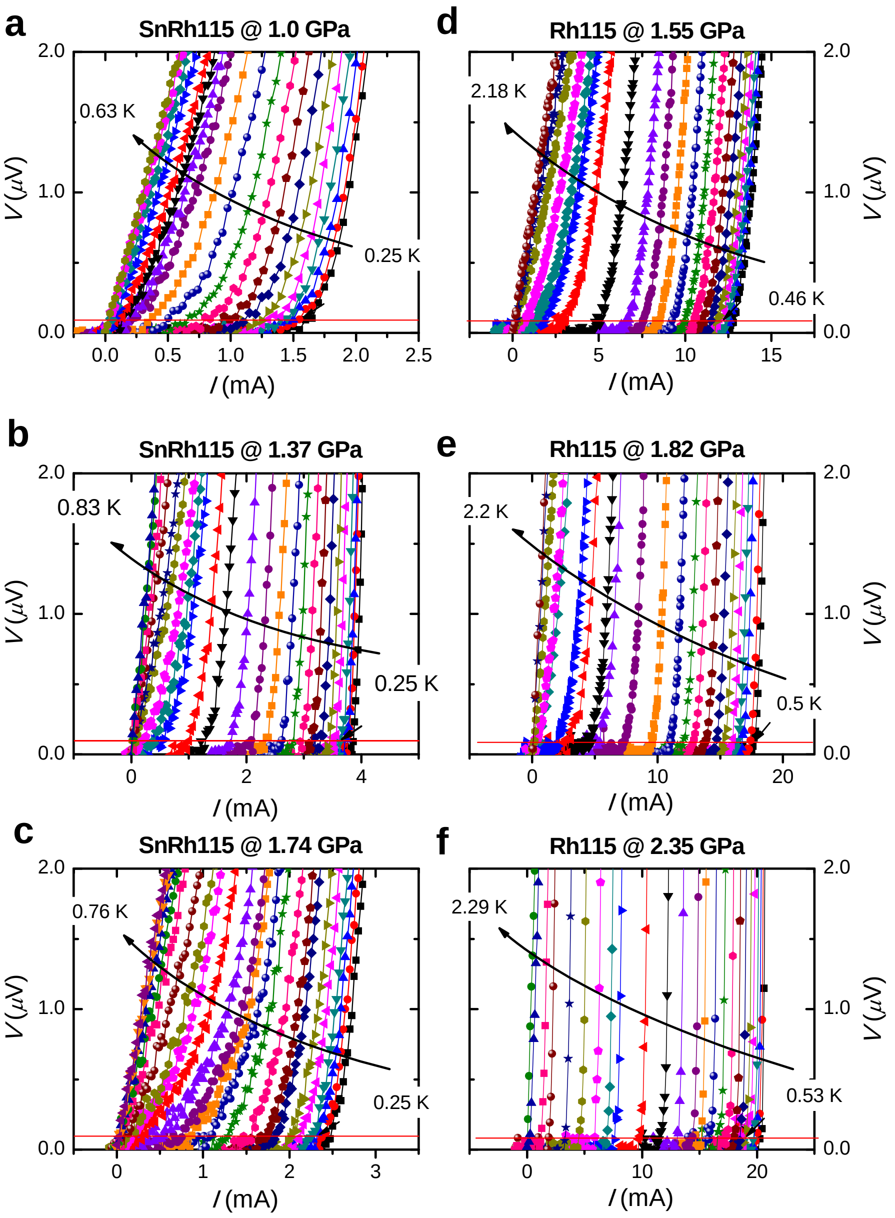

Supplementary Figure 2: Current – voltage curves at zero field for $CeRhSn_{0.22}In_{4.78}$ and $CeRhIn_5$ under various pressures. a-c, Current (I) – voltage (V) characteristic curves for the SnRh115 single crystal at pressures of 1.0, 1.37, and 1.74 GPa. **d-f,** $I - V$ characteristic curves for the Rh115 single crystal at pressures of 1.55, 1.82, and 2.35 GPa. The critical current (I_c) is determined using the V = 0.1 μV criterion that is indicated as a horizontal red line. Arrows indicate the temperature variation over which $I - V$ curves were measured.

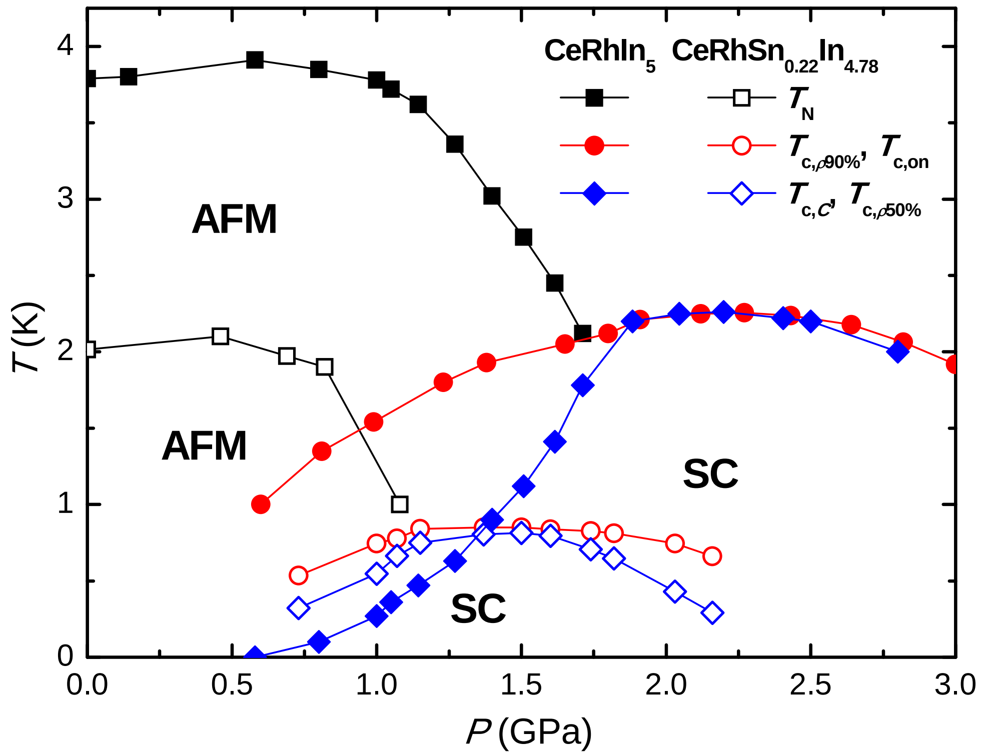

Supplementary Figure 3: Temperature-pressure phase diagrams for $CeRhIn_5$ and $CeRhSn_{0.22}In_{4.78}$ single crystals. Solid and open squares represent the antiferromagnetic (AFM) transition temperature (T_N) for Rh115 and SnRh115, respectively. Solid circles and solid diamonds describe the superconducting transition temperatures (T_c's) evaluated from resistivity ($T_{c,\rho 90\%}$) and specific heat ($T_{c,C}$) measurements on Rh115, respectively. Data at pressures above 2.35 GPa are adapted from Ref. [1]. Open circles ($T_{c,on}$) and open diamonds ($T_{c,\rho 50\%}$) are T_c of SnRh115 that were determined as T_c onset and 50% of the normal-state resistivity value at $T_{c,on}$, respectively.

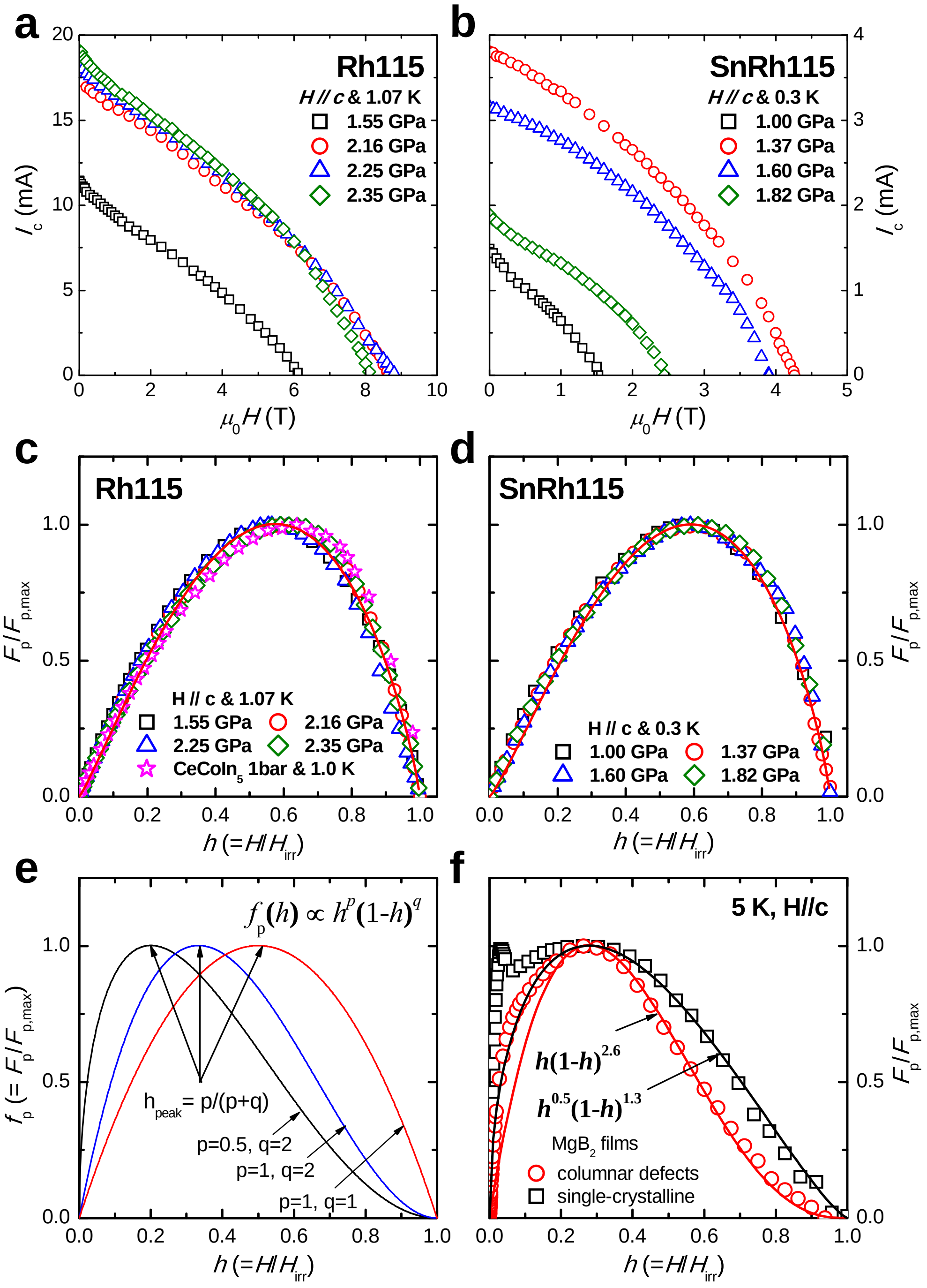

Supplementary Figure 4: Magnetic field dependences of critical current and normalized flux-pinning force for $CeRhIn_5$ and $CeRhSn_{0.22}In_{4.78}$. a, b show the critical current (I_c) as a function of magnetic fields for $CeRhIn_5$ (Rh115) and $CeRhSn_{0.22}In_{4.78}$ (SnRh115) under pressure, respectively. The flux pinning force (F_p) can be obtained from $I_c(H)$ using the relation $F_p = I_c \times \mu_0 H$. **c,** Reduced field ($h = H/H_{irr}$) dependence of the normalized flux-pinning force ($f_p = F_p/F_{p,max}$)

for Rh115 and $CeCoIn_5$, where H_{irr} is the irreversible field and $F_{p,max}$ is the maximum flux-pinning force. All $f_p(h)$ sets of data for Rh115 are well scaled by one curve irrespective of the pressure, and the $f_p(h)$ curve for $CeCoIn_5$ at ambient pressure is similar to Rh115 under pressure. **d,** $f_p(h)$ curves for SnRh115 also are well expressed by one curve regardless of the pressure. The similar scaling relations for $f_p(h)$ for all samples indicate that the main flux-pinning sources in the samples are the same [2,3]. **e**, Several commonly used scaling curves $f_p(h)$ are plotted against reduced field h [2,3]. The field dependence of the normalized flux-pinning force, $f_p(h)$, generally can be expressed by the relation $f_p(h) \propto h^p(1-h)^q$, and at low fields, the maximum flux-pinning force usually occurs at $h_{peak} = p/(p+q)$ in type-II superconductors with strong pinning. Phenomenologically, the fitting parameter p is close to 2 for strong pinning and $p \approx 1$ for weak pinning. Our Ce-based 115 compounds, which are very clean materials, show a scaling behaviour $f_p(h)$ with $h_{peak} \approx 0.6$, which is plotted as a red line in **a** and **b**, where the best results were obtained with $p = 1.15$ and $q = 0.85$ for Rh115 and $p = 1.2$ and $q = 0.83$ for SnRh115. **f**, For comparison, $f_p(h)$ is shown as a function of reduced field for single-crystalline MgB_2 thin films and MgB_2 films with a strong flux-pinning source of a columnar grain boundary.

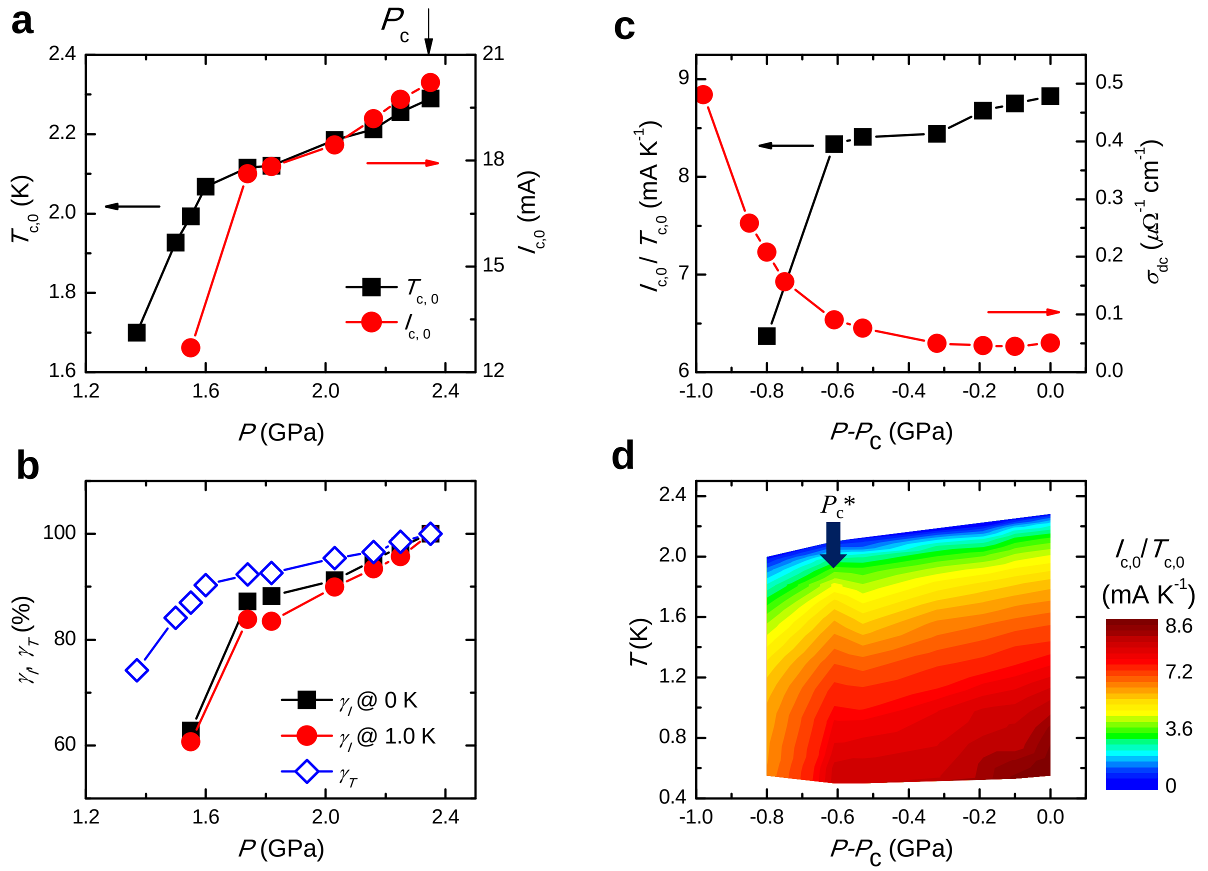

Supplementary Figure 5: Pressure evolution of the zero-field critical current in $CeRhIn_5$. a, Pressure dependences of $T_{c,0}$ and $I_{c,0}$ for Rh115, where $I_{c,0}$ is the value of I_c obtained from an extrapolation of data in Fig. 2a to zero Kelvin. **b,** Fractional variations in $I_{c,0}$ and $T_{c,0}$ for Rh115 under pressure. The fractions are defined as $\gamma_I \equiv I_c(T,P)/I_{c,0}(P_c) \times 100$ and $\gamma_T \equiv T_{c,0}(P)/T_{c,0}(P_c) \times 100$, where $I_{c,0}(P_c)$ is I_c extrapolated to zero temperature at P_c and $T_{c,0}(P_c)$ is the superconducting transition temperature at P_c. Values of γ_I are plotted as a function of pressure for measured or estimated $I_c(T,P)$ at 0 K (squares) and 1.0 K (circles). **c,** The ratio between the critical current and SC transition temperature, $I_{c,0}/T_{c,0}$, plotted together with the dc conductivity at T_c onset, σ_{dc}, as a function of the pressure difference P-P_c, where P_c = 2.35 GPa is the QCP. **d,** A contour plot of $I_{c,0}/T_{c,0}$ displayed in the temperature (T) and pressure (P-P_c) plane. The bold arrow marks P_c*, the boundary between a phase of coexisting antiferromagnetic and superconductivity and a solely superconducting phase, where there is a notable peak and a sudden enhancement in $I_c/T_{c,0}$ for Rh115.

Supplementary References

[1] Park, T. & Thompson, J. D. Magnetism and superconductivity in strongly correlated $CeRhIn_5$. *New. J. Phys.* **11**, 055062 (2009).

[2] Dew-Hughes, D. Flux pinning mechanisms in type-II superconductors. *Philos. Mag.* **30**, 293-305 (1974).

[3] Kramer, E. J. Scaling laws for flux pinning in hard superconductors. *J. Appl. Phys*. **44**, 1360-1370 (1973).
